# Supplementary material for: HIVprotI: an integrated web based platform for prediction and design of HIV proteins inhibitors
Source: J Cheminform. 2018 Mar 9;10:12. doi: 10.1186/s13321-018-0266-y (PMC5845081; doi:10.1186/s13321-018-0266-y)
Supplement: Supplementary file 2 — Additional file 2. Source code of HIVProtI web server. [file 13321_2018_266_MOESM2_ESM.zip › HIVprotI_Source-code/hivprotI/weka-3-6-6/doc/weka/gui/visualize/JComponentWriter.html]

JComponentWriter


---


|  |  |  |
| --- | --- | --- |
| **Overview** \| **Package** \| **Class** \| **Tree** \| **Deprecated** \| **Index** \| **Help** \| **Weka's home** | |  |
| **PREV CLASS**   **NEXT CLASS** | **FRAMES**    **NO FRAMES**     **All Classes** |  |
| SUMMARY: NESTED \| FIELD \| CONSTR \| METHOD | DETAIL: FIELD \| CONSTR \| METHOD |  |


---


## weka.gui.visualize Class JComponentWriter

```
java.lang.Object
  weka.gui.visualize.JComponentWriter
```

**Direct Known Subclasses:**: BMPWriter, JPEGWriter, PNGWriter, PostscriptWriter

---

``` public abstract class JComponentWriter extends java.lang.Object ```

This class takes any JComponent and outputs it to a file. Scaling is by
default enabled. Derived classes only need to override the following
methods:

- `getDescription()`
- `getExtension()`
- `generateOutput()`

**Version:**
:   $Revision: 7059 $

**Author:**
:   FracPete (fracpete at waikato dot ac dot nz)

**See Also:**: `setScalingEnabled(boolean)`

---

**Constructor Summary**

`JComponentWriter()`
  
          initializes the object

`JComponentWriter(javax.swing.JComponent c)`
  
          initializes the object with the given Component

`JComponentWriter(javax.swing.JComponent c,
java.io.File f)`
  
          initializes the object with the given Component and filename


| **Method Summary** | |
| --- | --- |
| `javax.swing.JComponent` | `getComponent()`             returns the component that is stored in the output format |
| `int` | `getCustomHeight()`             gets the custom height currently used |
| `int` | `getCustomWidth()`             gets the custom width currently used |
| `abstract  java.lang.String` | `getDescription()`             returns the name of the writer, to display in the FileChooser. |
| `abstract  java.lang.String` | `getExtension()`             returns the extension (incl. |
| `java.io.File` | `getFile()`             returns the file being used for storing the output |
| `boolean` | `getScalingEnabled()`             whether scaling is enabled or ignored |
| `boolean` | `getUseCustomDimensions()`             whether custom dimensions are to used for the size of the image |
| `double` | `getXScale()`             returns the scale factor for the x-axis |
| `double` | `getYScale()`             returns the scale factor for the y-axis |
| `void` | `setComponent(javax.swing.JComponent c)`             sets the component to print to an output format |
| `void` | `setCustomHeight(int value)`             sets the custom height to use |
| `void` | `setCustomWidth(int value)`             sets the custom width to use |
| `void` | `setFile(java.io.File f)`             sets the file to store the output in |
| `void` | `setScale(double x, double y)`             sets the scale factor - is ignored since we always create a screenshot! |
| `void` | `setScalingEnabled(boolean enabled)`             sets whether to enable scaling |
| `void` | `setUseCustomDimensions(boolean value)`             sets whether to use custom dimensions for the image |
| `void` | `toOutput()`             saves the current component to the currently set file. |
| `static void` | `toOutput(JComponentWriter writer, javax.swing.JComponent comp, java.io.File file)`             outputs the given component with the given writer in the specified file |
| `static void` | `toOutput(JComponentWriter writer, javax.swing.JComponent comp, java.io.File file, int width, int height)`             outputs the given component with the given writer in the specified file. |

**Methods inherited from class java.lang.Object**

`equals, getClass, hashCode, notify, notifyAll, toString, wait, wait, wait`

**Constructor Detail**

### JComponentWriter

```
public JComponentWriter()
```

:   initializes the object

---


### JComponentWriter

```
public JComponentWriter(javax.swing.JComponent c)
```

:   initializes the object with the given Component

    **Parameters:**: `c` - the component to print in the output format

---


### JComponentWriter

```
public JComponentWriter(javax.swing.JComponent c,
                        java.io.File f)
```

:   initializes the object with the given Component and filename

    **Parameters:**: `c` - the component to print in the output format: `f` - the file to store the output in


**Method Detail**

### setComponent

```
public void setComponent(javax.swing.JComponent c)
```

:   sets the component to print to an output format

    :   **Parameters:**: `c` - the component to print

---


### getComponent

```
public javax.swing.JComponent getComponent()
```

:   returns the component that is stored in the output format

    :   **Returns:**: the component to print

---


### setFile

```
public void setFile(java.io.File f)
```

:   sets the file to store the output in

    :   **Parameters:**: `f` - the file to store the output in

---


### getFile

```
public java.io.File getFile()
```

:   returns the file being used for storing the output

    :   **Returns:**: the file to store the output in

---


### getDescription

```
public abstract java.lang.String getDescription()
```

:   returns the name of the writer, to display in the FileChooser.
    must be overridden in the derived class.

    :   **Returns:**: the name of the writer

---


### getExtension

```
public abstract java.lang.String getExtension()
```

:   returns the extension (incl. ".") of the output format, to use in the
    FileChooser.
    must be overridden in the derived class.

    :   **Returns:**: the file extension

---


### getScalingEnabled

```
public boolean getScalingEnabled()
```

:   whether scaling is enabled or ignored

    :   **Returns:**: true if scaling is enabled

---


### setScalingEnabled

```
public void setScalingEnabled(boolean enabled)
```

:   sets whether to enable scaling

    :   **Parameters:**: `enabled` - whether scaling is enabled

---


### setScale

```
public void setScale(double x,
                     double y)
```

:   sets the scale factor - is ignored since we always create a screenshot!

    :   **Parameters:**: `x` - the scale factor for the x-axis: `y` - the scale factor for the y-axis

---


### getXScale

```
public double getXScale()
```

:   returns the scale factor for the x-axis

    :   **Returns:**: the scale scale factor for the x-axis

---


### getYScale

```
public double getYScale()
```

:   returns the scale factor for the y-axis

    :   **Returns:**: the scale scale factor for the y-axis

---


### getUseCustomDimensions

```
public boolean getUseCustomDimensions()
```

:   whether custom dimensions are to used for the size of the image

    :   **Returns:**: true if custom dimensions are used

---


### setUseCustomDimensions

```
public void setUseCustomDimensions(boolean value)
```

:   sets whether to use custom dimensions for the image

    :   **Parameters:**: `value` - whether custom dimensions are used

---


### setCustomWidth

```
public void setCustomWidth(int value)
```

:   sets the custom width to use

    :   **Parameters:**: `value` - the width to use **See Also:**: `m_UseCustomDimensions`

---


### getCustomWidth

```
public int getCustomWidth()
```

:   gets the custom width currently used

    :   **Returns:**: the custom width currently used **See Also:**: `m_UseCustomDimensions`

---


### setCustomHeight

```
public void setCustomHeight(int value)
```

:   sets the custom height to use

    :   **Parameters:**: `value` - the height to use **See Also:**: `m_UseCustomDimensions`

---


### getCustomHeight

```
public int getCustomHeight()
```

:   gets the custom height currently used

    :   **Returns:**: the custom height currently used **See Also:**: `m_UseCustomDimensions`

---


### toOutput

```
public void toOutput()
              throws java.lang.Exception
```

:   saves the current component to the currently set file.

    **Note:** this method calls `generateOutput()` which needs
    to be overriden in subclasses!

    :   **Throws:**: `java.lang.Exception` - if either the file or the component is `null`

---


### toOutput

```
public static void toOutput(JComponentWriter writer,
                            javax.swing.JComponent comp,
                            java.io.File file)
                     throws java.lang.Exception
```

:   outputs the given component with the given writer in the specified file

    :   **Parameters:**: `writer` - the writer to use: `comp` - the component to output: `file` - the file to store the output in **Throws:**: `java.lang.Exception` - if component of file are `null`

---


### toOutput

```
public static void toOutput(JComponentWriter writer,
                            javax.swing.JComponent comp,
                            java.io.File file,
                            int width,
                            int height)
                     throws java.lang.Exception
```

:   outputs the given component with the given writer in the specified file.
    If width and height are different from -1 then these sizes are used,
    otherwise the current ones of the component

    :   **Parameters:**: `writer` - the writer to use: `comp` - the component to output: `file` - the file to store the output in: `width` - custom width, -1 uses the component's one: `height` - custom height, -1 uses the component's one **Throws:**: `java.lang.Exception` - if component or file are `null`


---


|  |  |  |
| --- | --- | --- |
| **Overview** \| **Package** \| **Class** \| **Tree** \| **Deprecated** \| **Index** \| **Help** \| **Weka's home** | |  |
| **PREV CLASS**   **NEXT CLASS** | **FRAMES**    **NO FRAMES**     **All Classes** |  |
| SUMMARY: NESTED \| FIELD \| CONSTR \| METHOD | DETAIL: FIELD \| CONSTR \| METHOD |  |


---
